# Genomic, Network, and Phylogenetic Analysis of the Oomycete Effector Arsenal

**DOI:** 10.1128/mSphere.00408-17

**Published:** 2017-11-22

**Authors:** Jamie McGowan, David A. Fitzpatrick

**Affiliations:** Department of Biology, Genome Evolution Laboratory, Maynooth University, Maynooth, Co. Kildare, Ireland; Carnegie Mellon University

**Keywords:** comparative genomics, effectors, evolution, oomycota, secretome, similarity network

## Abstract

The oomycetes are a class of microscopic, filamentous eukaryotes within the stramenopiles-alveolate-*Rhizaria* (SAR) supergroup and include ecologically significant animal and plant pathogens. Oomycetes secrete large arsenals of effector proteins that degrade host cell components, manipulate host immune responses, and induce necrosis, enabling parasitic colonization. This study investigated the expansion and evolution of effectors in 37 oomycete species in 4 oomycete orders, including *Albuginales*, *Peronosporales*, *Pythiales*, and *Saprolegniales* species. Our results highlight the large expansions of effector protein families, including glycoside hydrolases, pectinases, and necrosis-inducing proteins, in *Phytophthora* species. Species-specific expansions, including expansions of chitinases in *Aphanomyces astaci* and *Pythium oligandrum*, were detected. Novel effectors which may be involved in suppressing animal immune responses in *Ap. astaci* and *Py. insidiosum* were also identified. Type 2 necrosis-inducing proteins with an unusual phylogenetic history were also located in a number of oomycete species. We also investigated the "RxLR" effector complement of all 37 species and, as expected, observed large expansions in *Phytophthora* species numbers. Our results provide in-depth sequence information on all putative RxLR effectors from all 37 species. This work represents an up-to-date *in silico* catalogue of the effector arsenal of the oomycetes based on the 37 genomes currently available.

**IMPORTANCE** The oomycetes are a class of microscopic, filamentous eukaryotes and include ecologically significant animal and plant pathogens. Oomycetes secrete large arsenals of effector proteins that degrade host cell components, manipulate host immune responses, and induce necrosis, enabling parasitic colonization. In this study, we catalogued the number and evolution of effectors in 37 oomycete species whose genomes have been completely sequenced. Large expansions of effector protein families in *Phytophthora* species, including glycoside hydrolases, pectinases, and necrosis-inducing proteins, were observed. Species-specific expansions were detected, including chitinases in *Aphanomyces astaci* and *Pythium oligandrum*. Novel effectors which may be involved in suppressing animal immune responses were identified in *Ap. astaci* and *Py. oligandrum*. Type 2 necrosis-inducing proteins with an unusual phylogenetic history were also located. This work represents an up-to-date *in silico* catalogue of the effector arsenal of the oomycetes based on the 37 genomes currently available.

## Abstract (precis)

The oomycetes are a class of microscopic, filamentous eukaryotes and include ecologically significant animal and plant pathogens. Oomycetes secrete large arsenals of effector proteins that degrade host cell components, manipulate host immune responses, and induce necrosis, enabling parasitic colonization. In this study, we catalogued the number and evolution of effectors in 37 oomycete species whose genomes have been completely sequenced. Large expansions of effector protein families in *Phytophthora* species, including glycoside hydrolases, pectinases, and necrosis-inducing proteins, were observed. Species-specific expansions were detected, including chitinases in *Aphanomyces astaci* and *Pythium oligandrum*. Novel effectors which may be involved in suppressing animal immune responses were identified in *Ap. astaci* and *Py. oligandrum*. Type 2 necrosis-inducing proteins with an unusual phylogenetic history were also located. This work represents an up-to-date *in silico* catalogue of the effector arsenal of the oomycetes based on the 37 genomes currently available.

## INTRODUCTION

The oomycetes are a class of diverse eukaryotic microorganisms which includes some of the most devastating pathogens of plants, mammals, fish, and fungi (1). Previously, they were thought to be fungi, due to the similar morphologies, ecological roles, and modes of nutrition and filamentous growth (2). Molecular analyses have placed the oomycetes into the *Stramenopiles-Alveolata-Rhizaria* (SAR) eukaryotic supergroup with close relationships to the diatoms and brown algae (3). Within the oomycete class, there are a number of highly diverse orders, including the *Saprolegniales*, *Peronosporales*, *Albuginales*, and *Pythiales*, that exhibit different lifestyles and can have either very specific or very broad host ranges.

**FIG 1:**
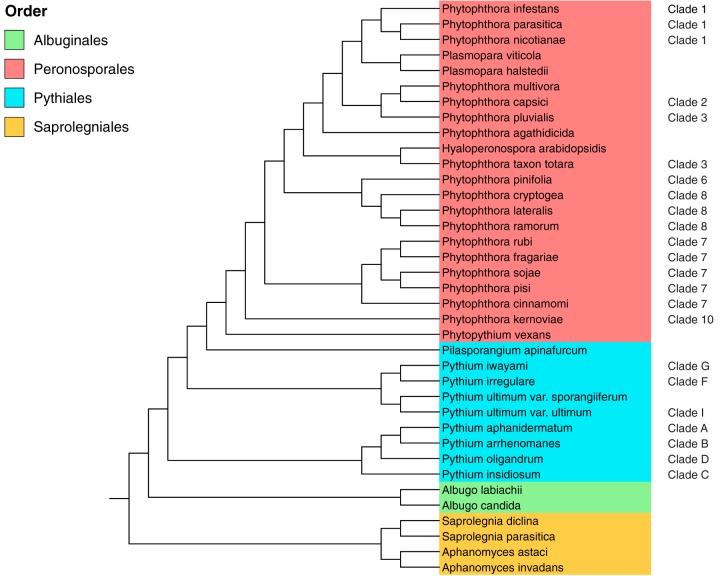
Phylogeny of the 37 oomycete species from 4 oomycete orders considered in this study. Data for *Phytophthora* clades as designated by Blair et al. (10) and *Pythium* clades as designated by de Cock et al. (19) are indicated in red and blue, respectively. (Adapted from reference 11.

More than 60% of known oomycetes are pathogens of plants (4) and have a devastating effect on many agriculturally important crops and ornamental plants. Members of the *Saprolegniales* order predominantly exhibit saprotrophic lifestyles and include the *Aphanomyces* animal and plant pathogens (5) as well as the fish-pathogenic *Saprolegnia* genus, known as “cotton molds” (6, 7). The *Peronosporales* order consists largely of phytopathogens and includes the hemibiotrophic genus *Phytophthora* (the “plant destroyers”). *Phytophthora* species include the notorious phytopathogen *Phytophthora infestans*, which is the causative agent of late potato blight, a disease reported to cause billions of dollars worth of damage worldwide annually (8). *Phytophthora* is the largest genus of the *Peronosporales* order and is divided into 10 phylogenetic clades (namely, clades 1 to 10) (9, 10) (Fig. 1). Also included in the *Peronosporales* are the genera *Hyaloperonospora* and *Plasmopara*, which are closely related to *Phytophthora* species (11) (Fig. 1). These two genera contain species that cause downy mildew in a number of economically important plants (12–14). In contrast to *Phytophthora* species, they are obligate biotrophs that are completely dependent on their host for survival. Other obligate oomycete biotrophs include the *Albugo* species (“white blister rusts”), which are members of the *Albuginales* order (15, 16) (Fig. 1). The *Pythiales* order includes the genus *Pythium*, members of which are necrotrophs that cause root rot in many terrestrial plants. Exceptions include *Pythium insidiosum*, a pathogen causing pythiosis in mammals (17), and *Pythium oligandrum*, a pathogen of other oomycetes and fungi (18). *Pythium* species are divided into 10 clades (namely, clades A to J) (19) (Fig. 1).

Oomycetes are notorious for secreting a large arsenal of effector proteins (20). Effectors facilitate infection by manipulating host cell components, exploiting host nutrients, triggering defense responses, and inducing necrosis (21). Oomycete effectors can be categorized into two classes (the apoplastic and cytoplasmic classes) depending on where they localize. Apoplastic effectors are secreted by the pathogens and exert their pathogenic activity in the host’s extracellular environment (22). Oomycete apoplastic effectors include a large number of hydrolytic enzymes which are involved in the degradation of host cell components, enabling penetration of host cells. These include cutinases, glycoside hydrolases (GHs), pectinases, and proteases, among other enzymes. Some oomycete species, such as *Phytophthora*, also encode members of extracellular toxin families such as necrosis-inducing proteins (NLPs) and Pcf family toxins (23). Host species are known to secrete protective proteases in an effort to degrade pathogen effectors; for example, P69B and P69C are subtilisin-like serine proteases secreted by tomatoes in response to *Phytophthora* species protease (24). To counteract this, *Phytophthora* species secrete protease inhibitors to block the host defense (25).

In contrast to apoplastic effectors, cytoplasmic effectors are secreted and translocated into the host cell, where they exhibit their pathogenic activity. Two types of cytoplasmic effectors dominate the oomycete secretome—"RxLR" effectors and Crinklers. RxLR effectors (RxLRs) are so named because they contain a highly conserved RxLR motif in their N-terminal domain (8, 26). This motif is followed by a downstream “EER” motif in many RxLRs. Studies have shown that the RxLR motif acts as a translocation signal, marking the protein for trafficking into the host cell (27). The mechanisms of the RxLR motif are thought to be similar to those of the “Pexel” translocation motif found in effectors of the malaria parasite *Plasmodium falciparum* (27–29). Some RxLRs can enter host cells in a manner independent of any additional pathogen-encoded machinery (30). RxLRs have been described as members of a rapidly evolving superfamily in which all members are related and share a common ancestor (31). They have very conserved N termini and divergent C termini, although conserved WY folds have been observed in some (8, 32). Genes encoding RxLR effectors are mainly found in gene-sparse regions of the genome which contain a high frequency of transposons (33). This could account for the rapid evolution of RxLR effectors. Large expansions of RxLR effectors have been observed in *Phytophthora* species, with some species reportedly encoding several hundred putative RxLRs (8, 26).

Other well-characterized oomycete effectors include Crinkler proteins (CRNs), named for their crinkling and necrosis-inducing activity, which are composed of a highly conserved N-terminal domain containing a signal peptide and an “LxLFLAK” motif which mediates translocation into the host cell (34). The end of the N terminus is marked by a highly conserved “HVLVxxP” motif, which separates the N terminus and C terminus. They are modular, rapidly evolving proteins that consist of a diverse collection of C-terminal domains (8, 23). The CRNs of some oomycetes carry a modified version of the “LxLFLAK” motif (15). CRNs have been reported to localize to, target, and accumulate in host nuclear components (34, 35). CRNs are thought to be a more ancient class of cytoplasmic effectors than RxLRs, as they have been found to be distributed across a wide range of oomycete orders (23), including *Albuginales* (15, 16), *Peronosporales* (8, 26, 36), and *Pythiales* (37, 38).

In this report, we catalogue the effector repertoire among 590,896 protein coding genes from 37 publically available genome sequences for the oomycete class, including *Albugo*, *Aphanomyces*, *Hyaloperonospora*, *Phytophthora*, *Phytopythium*, *Pilasporangium*, *Plasmopara*, *Pythium*, and *Saprolegnia* species (Table 1). Numerous bioinformatic techniques were employed to identify and catalogue putative proteins which may be involved in pathogenesis. A mix of network and phylogenetic methods was utilized to analyze their evolutionary history. Our results have identified novel effector families that appear to be unique to particular oomycete lineages, including *Ap. astaci* proteins, which might have the potential to cleave immunoglobulin. Consistent with previous studies, we have identified a significant expansion of effectors in *Phytophthora* species, including glycoside hydrolases and necrosis-inducing proteins. We have detected expansions of chitin degrading enzymes in *Ap. astaci* and *Py. oligandrum*. We have also identified multiple type 2 necrosis-inducing proteins in a number of oomycete species.

**TABLE 1:**
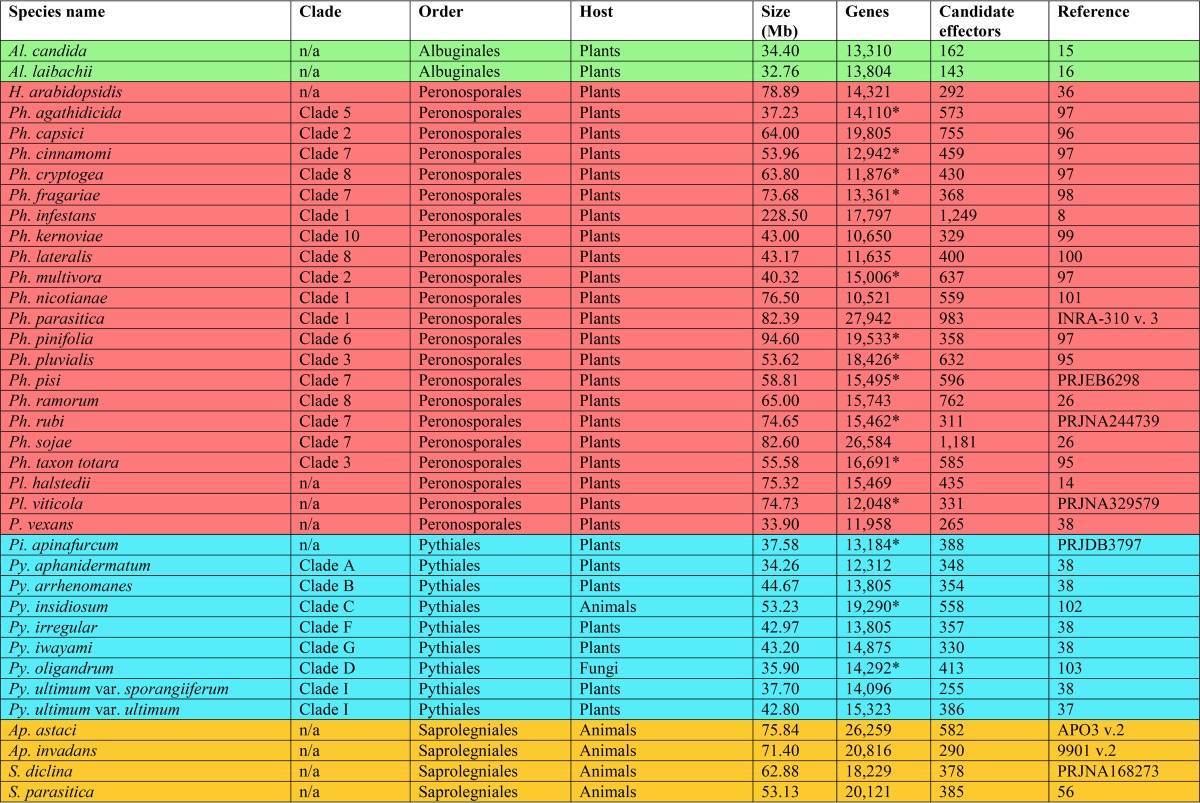
Taxonomic and genomic information for the 37 oomycete species analyzed here^a^

a Protein counts manually generated from assembly data are marked with an asterisk. References are to the genome publications where possible or otherwise to NCBI BioProject identifiers or to the Broad Institute strain identifier and assembly version. Species are colored by order as follows: green, *Albuginales*; red, *Peronosporales*; blue, *Pythiales*; orange, *Saprolegniales*. (Adapted from reference 11).

## RESULTS AND DISCUSSION

### The oomycete secretome.

Oomycete pathogens secrete effector proteins and degradative enzymes to facilitate host colonization through altered physiology (38). Using the available proteome data, we undertook *in silico* prediction analyses to determine the number of proteins in each species that may be secreted. Our data set consisted of the predicted proteomes of 37 oomycete species. This included 18 *Phytophthora*, 8 *Pythium*, 2 *Albugo*, 2 *Aphanomyces*, 2 *Plasmopara*, 2 *Saprolegnia*, 1 *Hyaloperonospora*, 1 *Pilasporangium*, and 1 *Phytopythium* species (Table 1).

Proteins predicted to contain signal peptides were located using SignalP v3. SignalP v3 was chosen over earlier and later versions of the software as previous studies have found v3 the most sensitive in detecting oomycete signal peptides (39). Proteins that contained a transmembrane domain after the signal peptide cleavage were discarded as these proteins are not likely to be secreted and are instead retained in the plasma membrane.

A previous analysis of 13 stramenopiles (including 11 species in our data set) by Adhikari et al. found that between 6.19% and 10.34% of the proteins in each species were secreted (38). Our analysis showed that of the 590,896 proteins tested, 5.25% (30,996) are predicted to be secreted, from a relative low of 2.11% (291) in the obligate biotroph *Al. laibachii* to a relative high of 7.93% (834) in the necrotroph *Ph. nicotianae* (see Table S1 in the supplemental material). We observed that in all cases, the percentages of secreted proteins in our analysis differed from what was observed in the analysis by Adhikari et al. These differences cannot be accounted for by differences in the number of proteins per species as the numbers were consistent between the two analyses, as were the methodologies used. For example, Adhikari et al. predicted that 10.34% of the *Ph. ramorum* genome is secreted compared to 7.65% in our analysis (Table S1). Similarly, there are large discrepancies between the percentages of secreted proteins for *Ph. sojae* (10.24% versus 6.24%) and for *Py. irregular* (6.95% versus 5.03%) and the largest discrepancy was seen in the comparisons for *H. arabidopsidis* (8.81% versus 3.68%). For transparency, all scripts used to annotate secreted proteins are publically available (see Materials and Methods).

10.1128/mSphere.00408-17.4 TABLE S1  Overall counts of predicted secreted oomycete proteins per species. Signal peptides were predicted using SignalP v3. Download TABLE S1, XLSX file, 0.04 MB. Copyright © 2017 McGowan and Fitzpatrick. 2017 McGowan and Fitzpatrick This content is distributed under the terms of the Creative Commons Attribution 4.0 International license.

Our results show there is significant a difference (*P* < 0.01 [chi square test]) between two of the closely related *Saprolegniales* species (*Ap. astaci* and *Ap. invadans*) in the number of predicted proteins, where 4.26% (1,119 predicted proteins) of the *Ap. astaci* proteome is predicted to be secreted compared to 2.93% (609 predicted proteins) in *Ap. invadans* (Table S1). The predicted proteomes for those two species differ in size (26,529 versus 20,816 predicted proteins), indicating that an expansion in secreted proteins is partially responsible for the differences observed in the sizes of the proteomes.

Overall, the correlation between the number of predicted secreted proteins and the overall number of proteins per species was shown to be moderate (*R*^2^ = 0.455) and significant (*P* < 0.00001 [Pearson correlation test]). The correlation between the number of predicted secreted proteins and genome size was shown to be weak (*R*^2^ = 0.1065), however, and not significant (*P* > 0.05).

### Secretome enrichment analysis.

We have documented which biological functions are enriched in the predicted secretomes of individual species. This was achieved by comparing the frequency of Gene Ontology (GO) terms and Pfam domains in the secretome to the nonsecreted proportion of the proteome using the Fisher exact test corrected for false-discovery rate (FDR) (40). InterProScan was used to functionally annotate all proteins with GO terms and Pfam domains (41).

**FIG 2:**
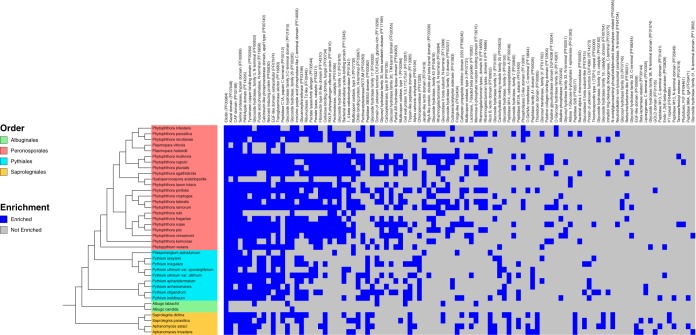
Heat map of enriched Pfam terms in oomycete secretomes. Terms are ordered with respect to the number of the times that they were observed. Only terms listed for two or more species are shown. Terms that were statistically enriched are colored blue in the heat map. Gray coloring indicates that the term was not enriched.

Comparing the putative secretomes of the 37 species in this analysis, we saw that the number of Pfam domains enriched in the secretome relative to the nonsecreted portion of the proteome varied from a low of 2 domains in *Albugo candida* to a high of 56 in *Ap. astaci* (Table S2). The enrichment analysis showed that the elicitin Pfam domain (PF00964) is enriched in all 37 species (Fig. 2 and Table S2). Elicitins are structurally conserved extracellular proteins in *Phytophthora* and *Pythium* species (37). They are known to bind lipids and sequester sterols from plants, thereby overcoming the inability of *Phytophthora* and *Pythium* species to synthesize sterols (42). Similarly, the serine protease inhibitor Kazal-like domain (PF07648) is enriched in 33 of the 37 species (Fig. 2 and Table S2). The Kazal-like domain has been implicated in the infection process of *Phytophthora* species and acts as an apoplastic effector (24). Another widely distributed domain that is enriched in a large number of oomycete secretomes (33 of 37) is that corresponding to the cysteine-rich secretory proteins, antigen 5, and pathogenesis-related 1 proteins (CAP) (Fig. 2 and Table S2). CAP-domain-containing secreted proteins are produced by nonvertebrate eukaryotes and prokaryotes and have been implicated in both virulence and immunity functions (43, 44); however, little is currently known about the molecular mode of action of such proteins. Of the 37 species analyzed, 31 showed enrichment for chymotrypsin (PF00089), which most probably has a role to play in extracellular proteolysis. Similarly, 30 of the 37 species showed enrichment for the PAN/Apple domain (PF14295). Previous analyses had shown that carbohydrate-binding module (CBM)-containing proteins that recognize and bind saccharide ligands from *Ph. parasitica* are associated with two PAN/Apple domains. The PAN/Apple domain is known to interact with both proteins and carbohydrates (45). Knockdown of *Ph. parasitica* CBM affects adhesion to cellulose subtrates, including plant cell walls (46). Domains involved in the possible degradation of plant cell walls were also found to be enriched in the secretomes of many of the species investigated. For example, pectin degradation (PF00544, PF03283, and PF03211), glycoside hydrolases (PF00933, PF00915, PF00295, PF17189, and PF02055) and cellulose binding (PF00374) are all enriched across a range of species (Fig. 2 and Table S2). Well-known effectors, including necrosis-inducing protein (PF05630), are found to be enriched in *Phytophthora* and *Pythiales* species, while the RxLR phytopathogen domain (PF16810) is enriched in the secretome of the majority of *Phytophthora* species (Fig. 2 and Table S2).

10.1128/mSphere.00408-17.5 TABLE S2  Pfam domains and GO terms enriched in the secretome of cognate oomycete genomes. Only domains or terms significantly enriched are shown. Download TABLE S2, XLSX file, 0.3 MB. Copyright © 2017 McGowan and Fitzpatrick. 2017 McGowan and Fitzpatrick This content is distributed under the terms of the Creative Commons Attribution 4.0 International license.

With respect to GO term secretome enrichment within our data set, we observed results that corroborated our Pfam enrichment analysis. For example, enriched GO terms associated with plant cell wall degradation such as pectin activity (GO: 0030570 and GO: 0030599), cellulose activity (GO: 0030248 and GO: 0008810), polygalacturonase activity (GO: 004650), glucan catabolism (GO: 0009251), cellulose catabolism (GO: 0030245), cellulose metabolism (GO: 0030243), and carbohydrate binding (GO: 0030246) are widely distributed. Cell wall organization (GO: 0071555), modification (GO: 0042545), and biogenesis (GO: 0071554) are all enriched in the majority of *Phytophthora* species (see Table S2 and Fig. S1 in the supplemental material). Hydrolase activities acting on glycosyl bonds (GO: 0004553, GO: 0016787, and GO: 0016798) were found to be enriched in all 37 species (Table S2 and Fig. S1). Other terms that are ubiquitously enriched include defense response (GO: 0006952), pathogenesis (GO: 0009405), interspecies interactions (GO: 0044419), and multiorganism processes (GO: 0051704) (Table S2 and Fig. S1).

10.1128/mSphere.00408-17.1 FIG S1  Heat map of enriched GO terms in oomycete secretomes. Terms are ordered with respect to the number of the times that they were observed. (A) Biological process. (B) Molecular function. Download FIG S1, PDF file, 0.8 MB. Copyright © 2017 McGowan and Fitzpatrick. 2017 McGowan and Fitzpatrick This content is distributed under the terms of the Creative Commons Attribution 4.0 International license.

Previous researchers have undertaken similar analyses of some of the species within our data set, including *Ph. infestans* (47) and six *Pythium* species (38). We found broad agreement between our results and those previously reported for the *Ph. infestans* analysis. For example, we also observed enrichment in GO terms associated with carbohydrate metabolic processes (GO: 0005975 and GO: 0016052), sugar metabolism (GO:0006040), sugar binding (GO: 0030246), sugar modification (GO: 0008810, GO: 0004650, GO: 0030570, and others), pathogenesis (GO: 0009405), defense response (GO: 0006952) proteolysis (GO: 0006508), and serine peptidase activity (GO: 0004252 and GO: 0008236) (Table S2). Similarly, we also observed Pfam domains associated with pectin degradation (PF03283, PF00544, and PF03211), elicitins (PF00964), Kazal-type domains (PF07648), and necrosis-inducing protein (PF05630) (Table S2). With respect to our results and those previously reported for *Pythium* species, the level of agreement is not as strong. We did observe enrichment in GO terms associated with pathogenesis, proteolysis, carbohydrate metabolic process (GO: 0006508), hydrolyase activity (GO: 0004553), and glycosyl hydrolyase activity (GO: 16798) but failed to detect enrichment in terms such nucleotide binding (GO: 000166), integral to membrane (GO: 0016021), transmembrane transport (GO: 0055085), and RNA processing (GO: 0006396) as previously reported (38).

### The oomycete effector arsenal.

We set out to investigate the abundance of effectors in oomycete species. The list of effectors considered in our analysis (Table S3) is based on a number of previous studies which described pathogenic proteins from oomycete species, including *Plasmopara* (14), *Phytophthora* (26), and *Pythium* (37) species. Any protein identified as having a pathogenicity-related domain was classified as a putative effector. These were combined with our secretome analysis to determine whether or not the effector was predicted to be secreted. The overall effector content of each species was quantified to detect expansions. The following counts excluded RxLR effectors as they are treated in more depth in a following section.

10.1128/mSphere.00408-17.6 TABLE S3  Overall counts of oomycete proteins with pathogenicity-related domains. Proteins were functionally annotated using InterProScan 5. Counts of secreted oomycete proteins with pathogenicity-related domains are also given. Download TABLE S3, XLSX file, 0.1 MB. Copyright © 2017 McGowan and Fitzpatrick. 2017 McGowan and Fitzpatrick This content is distributed under the terms of the Creative Commons Attribution 4.0 International license.

In total, 13,751 proteins were identified as having domains that could be implicated in pathogenicity (Table S3). Overall, 6,250 (~45%) of these were predicted to be secreted by SignalP (Table S3). Our results show that *Phytophthora* proteomes generally contain the highest frequency of effectors, with *Ph. sojae* (733 effectors) and *Ph. infestans* (646 effectors) possessing the largest arsenals of effectors, representing 2.76% and 3.63% of their total proteomes, respectively (Table S3). *Albugo* species were found to contain the smallest number of effectors. A trend was identified whereby hemibiotrophic species, such as *Phytophthora*, typically possess more effectors than saprotrophic species (members of the *Saprolegniales* order) and necrotrophs, such as *Pythium* species. Three species of obligate biotrophs, including *Al. candida*, *Al. laibachii*, and *H. arabidopsidis*, feature the lowest numbers of effectors, 156, 131, and 197, respectively (Table S3). Exceptions to this trend exist, most notably, *Ap. astaci* (554 effectors), *Py. insidiosum* (494 effectors), and *Py. oligandrum* (359 effectors), all of which contain large repertoires of effectors compared to other closely related oomycete species with similar lifestyles. For example, *Ap. astaci* was found to contain 554 effectors, in comparison to the closely related *Ap. invadans* species, which contained 272 (Table S3). A number of oomycete effectors are discussed in more detail in the following sections.

### Necrosis-inducing proteins (NLPs).

Necrosis-inducing proteins (NLPs) are apoplastic effectors found in bacteria, fungi, and oomycetes (48). The mechanisms by which NLPs act are not fully understood, but they are known to induce necrosis, trigger ethylene accumulation, and elicit immune responses in dicotyledons (49). A number of NLPs have previously been reported to be noncytotoxic but to act instead as microbe-associated molecular patterns (MAMPs) which are recognized by the plant hosts and result in the activation of the plant immune system (50).

Our InterProScan analysis detected 771 proteins with signatures of NLPs; 499 (67%) of these NLPs were predicted to contain signal peptides by SignalP (Table S3). Our results show that NLPs are absent from *Albugo*, *Aphanomyces*, and *Saprolegnia* species but are present in all *Peronosporales* and *Pythiales* species. Most *Pythium* species have fewer than seven copies. *Py. insidiosum* is the only *Pythium* species in our data set to lack NLPs. NLPs are highly expanded in *Phytophthora* species. In particular, *Ph. ramorum* has 69 copies, *Ph. parasitica* has 74 copies, and *Ph. sojae* has 80 copies (Table S3).

NLPs can be divided into two types: type 1 NLPs and type 2 NLPs (51). The two types share a conserved amino acid motif, “GHRHDWE.” They are distinguished by the presence of pairs of cysteine residues. Type 1 NLPs have one pair of conserved cysteines, while type 2 NLPs have two pairs of conserved cysteine residues (49). Each pair of cysteines could potentially form disulfide bridges, providing additional stability. Type 1 NLPs are found in bacteria, fungi, and oomycetes. Type 2 NLPs have been located in bacteria and fungi but were originally thought to be absent from oomycetes (49, 51). However, work by Horner et al. has shown that *Py. oligandrum* contains a type 2 NLP with similarity to a homolog from the proteobacterial plant pathogen *Pectobacterium atrosepticum* (52).

**FIG 3:**
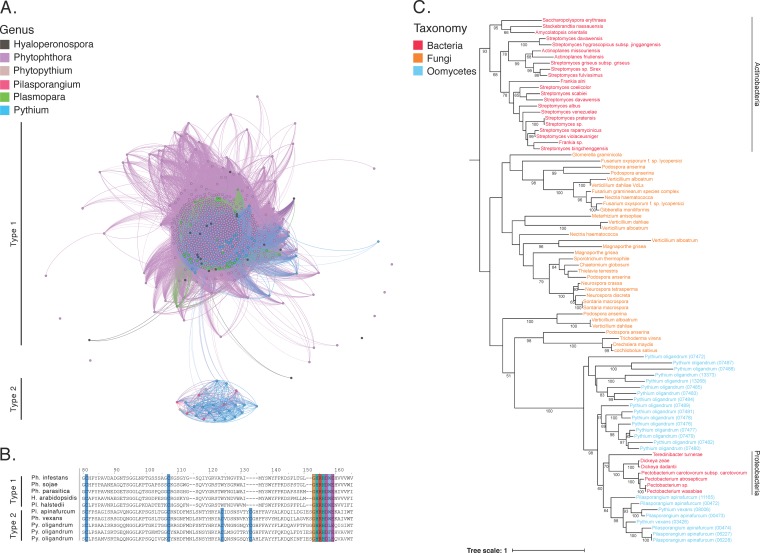
Analysis of oomycete necrosis-inducing proteins (NLP). (A) Homology network of 771 oomycete NLPs. Identified type 2 NLPs are arranged in a small, strongly connected cluster of 25 proteins from *Phytopythium vexans*, *Pilasporangium apinafurcum*, and *Pythium oligandrum*. (B) Multiple-sequence alignment of five type 1 NLPs and five type 2 NLPs. The two types share a conserved “GHRHDWE” NLP motif. Type 2 NLPs have an additional pair of cysteine residues. (C) Maximum-likelihood phylogeny of 87 NLPs containing 29 bacterial proteins, 33 fungal proteins, and 25 oomycete proteins. Bootstrap values greater than 50% are shown.

To further investigate the oomycete NLPs in our data set, we constructed a homology network of all 771 NLPs in our data set (Fig. 3A). An interactive version of the network is available online at https://oomycetes.github.io. In the network, the degree of a node is the number of edges it has connecting it to other nodes; therefore, the degree is the number of homologs that the protein has in our network. The overall NLP network (Fig. 3A) has an average degree value of 522.5, revealing that many of these NLPs are homologous to each other. 740 NLPs are grouped in a large, dense cluster (Fig. 3A). The network included six singletons that did not share significant sequence similarity with any other protein in the network. They included one *H. arabidopsidis*, one *Ph. cryptogea*, one *Ph. kernoviae*, and three *Ph. parasitica* proteins. Interestingly, a smaller cluster of 25 NLPs was identified (Fig. 3A). Only five of these proteins were homologous to other proteins outside this cluster; this lack of homology is illustrated by the few edges seen between both clusters (Fig. 3A). The 25 proteins included 17 *Py. oligandrum*, 6 *Pilasporangium apinafurcum*, and 2 *Phytopythium vexans* putative NLPs. One of the *Py. oligandrum* proteins is an ortholog of the type 2 NLP (GenBank accession number EV243877) previously reported by Horner et al. (52). A multiple-sequence alignment of a selection of proteins from both clusters was carried out (Fig. 3B). Inspection of this alignment revealed no significant sequence similarity between the two clusters except for the presence of the conserved “GHRHDWE” motif and conserved cysteine residues (Fig. 3B). The NLPs from the large cluster contained two conserved cysteine residues, indicating that they are type 1 NLPs. Proteins from the smaller cluster of 25 proteins contained four conserved cysteine residues, indicating that they are type 2 NLPs. Apart from the shared NLP motif and conserved cysteine residues, there is no significant sequence similarity between the two types of NLPs.

We set out to further investigate the evolutionary history of the putative oomycete type 2 NLPs. BLASTp searches of the 25 type 2 NLPs against the NCBI databases revealed top hits with proteobacterial species. To reconstruct the evolutionary history of these proteins, we used the 25 type 2 NLPs as bait sequences in a BLASTp homology search (*E* value cutoff of 10^−20^) against a local database of 8,805,033 proteins, with broad taxon sampling across prokaryotes and eukaryotes (53). This search identified 87 homologous proteins, including 25 oomycete proteins (included in our data set), 33 fungal proteins, and 29 bacterial proteins. We aligned and manually edited the resulting homologs to give a final alignment of 460 amino acids and generated a maximum-likelihood phylogeny with PhyML using the Whelan and Goldman (WAG) model of substitution (Fig. 3C). This phylogeny places all fungal proteins in a single clan (41% bootstrap support). All oomycete proteins were located in a single clan with 100% bootstrap support (Fig. 3C). Within this large oomycete-containing clan, there was also a sister group clan of *Proteobacteria* proteins with 70% bootstrap support (Fig. 3C). There was also a completely separate actinobacterial clan with 93% bootstrap support (Fig. 3C).

The phylogenetic distribution of these type 2 NLPs is interesting. All of the fungal homologs are from the subphylum *Pezizomycotina*. Two bacterial phyla are represented, the *Actinobacteria* and the *Proteobacteria*. However, the *Proteobacteria* proteins are inferred to be more closely related to their oomycete homologs than to the actinobacterial homologs (Fig. 3C). One possible scenario is that horizontal gene transfer (HGT) has occurred during the evolutionary history of these proteins. However, due to the patch phyletic distribution, we cannot confidently infer the direction of gene transfer or indeed whether HGT has definitely occurred.

### Immunoglobulin A peptidases.

Immunoglobulin A peptidases represent a family of hydrolytic enzymes that cleave immunoglobulin A (IgA) and have been implicated as important virulence factors in bacterial infections of humans (54). InterProScan analysis revealed 40 oomycete proteins containing “IgA peptidase M64” domains (Table S3), 21 (53%) of which were predicted to contain signal peptides (Table S3). Expansions of these proteins are present in the genomes of both *Ap. astaci* and *Py. insidiosum*. Ten proteins containing this domain were found in *Ap. astaci* (6 with signal peptides) and 6 in *Py. insidiosum* (4 with signal peptides) (Table S3). Other *Pythium* species in our data set contained between one and five copies of the protein. No proteins with this domain were found in any *Albugo*, *Phytophthora*, *Plasmopara*, or *Saprolegnia* species (Table S3). Therefore, within our data set, IgA peptidases are unique to *Aphanomyces* species and the *Pythiales* order.

**FIG 4:**
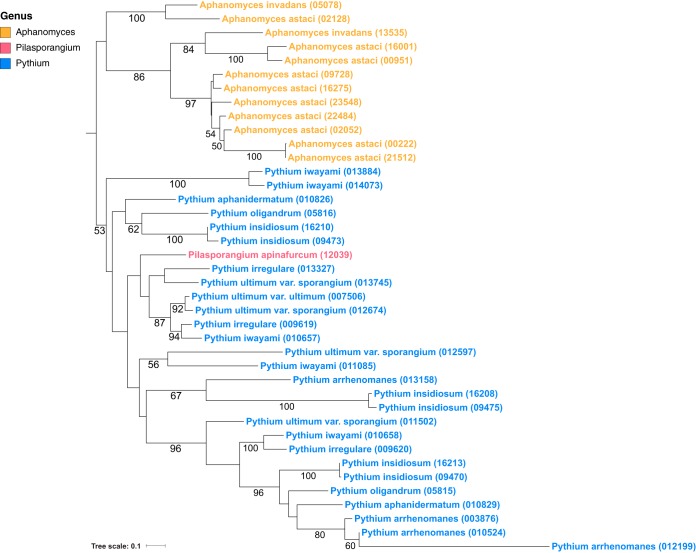
Maximum-likelihood phylogenetic reconstruction of oomycete IgA peptidases. Bootstrap values greater than 50% are shown.

We aligned and manually curated all 40 IgA peptidase-containing proteins using MUSCLE and JalView, respectively, to give a final alignment of 770 amino acids. A maximum-likelihood phylogeny of all IgA peptidase-containing proteins was generated using a WAG substitution model and 100 bootstrap replicates (Fig. 4). Our phylogenetic reconstruction shows multiple species-specific duplication events which have led to the expansion of IgA peptidases in *Ap. astaci* and *Py. insidiosum* (Fig. 4). The IgA peptidases are grouped into two separate monophyletic clades. The first contains all *Pythiales* homologs, while the second contains all *Aphanomyces* homologs (Fig. 4).

*Ap. astaci* and *Py. insidiosum* are pathogens of crayfish and mammals, respectively (17, 55). It is tempting to speculate that these proteins possibly provide a defense mechanism for these species, allowing them to suppress the immune response of their animal hosts. However, it should be noted that a number of *Pythium* species with plant hosts were also shown to contain multiple copies of IgA peptidases, including five in *Py. iwayami* and four in both *Py. arrhenomanes* and *Py. ultimum* var. *sporangiiferum* (Table S3).

### Glycoside hydrolases.

Glycoside hydrolases (GH), or glycosyl hydrolases, are hydrolytic proteins that cleave glycosidic bonds in complex sugars. They can be used to break down cellular components, for example, the cell membrane and cell wall. We set out to investigate the extent of expansions within the members of GH families across oomycete species. A total of 4,521 proteins with GH domains were located in our InterProScan analysis (Table S3 and S4). Expansions of GHs have been observed previously in oomycetes (38, 56). These were distributed across 47 different GH families. SignalP predicted secretion of 1,928 (42.6%) GHs. *Phytophthora* species possess the highest number of GH proteins, with an average of 162, followed by *Aphanomyces* and *Saprolegnia* species, with averages of 106 and 98, respectively (Table S4). *Al. candida* and *Al. laibachii* feature smaller sets of GH proteins, with fewer than 60 each.

10.1128/mSphere.00408-17.7 TABLE S4  Counts of oomycete glycoside hydrolase family proteins. InterProScan 5 was used to functionally annotate proteins and identify glycoside hydrolases. In total, 4,521 glycoside hydrolases, distributed across 47 glycoside hydrolase families, were identified. Corresponding Pfam IDs are marked. Download TABLE S4, XLSX file, 0.1 MB. Copyright © 2017 McGowan and Fitzpatrick. 2017 McGowan and Fitzpatrick This content is distributed under the terms of the Creative Commons Attribution 4.0 International license.

A number of GH families were found to be species or genus specific. For example, GH families 4, 26, 29, 42, and 48 are restricted to *Ph. kernoviae* whereas GH families 8, 24, and 44 are unique to *Ph. rubi*. Similarly, members of GH family 36 are found only in *Ph. kernoviae* and *Ph. rubi*. Members of GH families 20 and 27 are found only in members of the *Saprolegniales* order. GH families 18, 19, and 48 have known chitinase activity (57). Expansions of these families were revealed in *Ap. astaci*, *Py. oligandrum*, and *Saprolegnia* species. They are investigated in more detail below (see also Table S4).

### Chitinases.

Chitin is the second-most-abundant biopolymer in nature and is an important structural component of invertebrate exoskeletons and fungal cell walls (58). Chitinases are enzymes that degrade chitin. In fungi, chitinases play an important role in hyphal growth, spore germination, and cell wall remodeling (59). Oomycete cell walls, however, contain no or very small amounts of chitin (60). Thus, it would appear that chitinases encoded by oomycete species play roles primarily in the degradation of exogenous chitin, for example, in the breakdown of chitin in host cell walls.

Several GH families are known to have chitinase activity (57). These include GH families 18, 19, 23, and 48. A total of 281 proteins in our data set were reported to have putative chitinase activity in our InterProScan analysis; 112 (40%) of these are predicted to be secreted (Table S3). The chitinases identified were spread across three GH families, 18, 19, and 48. No members of GH family 23 were located in our data set. Only one protein was identified as a member of GH family 48; it belonged to *Ph. kernoviae*. However, it was not predicted to be secreted.

A large expansion of 50 chitinases was detected in *Ap. astaci* (Table S3). This is significantly more than the expansion seen with any other oomycete; 38 of these proteins belong to GH family 18, and the remaining 12 belong to GH family 19 (Table S4). *Ap. astaci* is a pathogen of crayfish, growing on and within the crayfish cuticle (55), which is composed of chitin. This expansion of chitinases may reveal a successful adaption of *Ap. astaci* to its crayfish host, allowing the pathogen to penetrate through the chitin layers of crayfish cuticles. In agreement with previous work (56), our analysis also revealed large numbers of chitinases in the genome of *S. diclina* and *S. parasitica* (25 and 23, respectively) (Table S3). Additionally, large numbers of chitinases were located in *Al. laibachii* and *Py. oligandrum*. A total of 16 chitinases were identified in *Al. laibachii* and 15 in *Py. oligandrum* (Table S3). This is particularly significant for *Py. oligandrum*, which is a pathogen of fungi (18). Some fungal cell walls are composed of up to 20% chitin (61). Possessing a large repertoire of chitinase enzymes may prove useful for the pathogen for breaking down host fungal cells. Other oomycetes in the data set typically had fewer than 10 chitinases.

We constructed a homology network of all 281 oomycete chitinases in our data set to investigate their evolutionary history (Fig. S2). An interactive version of the network is available online at https://oomycetes.github.io. In the networks, nodes represent proteins and edges represent sequence similarity between two proteins. Our network consists of a number of disconnected clusters (no edges/homology to other clusters). We identified one singleton (a node with no edges, i.e., a protein with no homologs), the GH family 48 protein belonging to *Ph. kernoviae*. All GH family 19 proteins were placed into a single, strongly connected cluster (Fig. S2). No *Albugo* proteins were found in this cluster. GH family 18 proteins were divided into five clusters. *Saprolegnia* proteins dominated the network; in particular, proteins of *Ap. astaci* made up almost 18% of the network. Thus, our network analysis shows that oomycete chitinases are divided into a number of subclasses that are not homologous. This is consistent with the division of chitinases into 5 classes (62).

10.1128/mSphere.00408-17.2 FIG S2  Homology network of 281 oomycete chitinases. Each protein is represented by a node. An edge joining two nodes represents sequence similarity shared by the two proteins (*E* value cutoff of 10^−10^). All members of GH family 19 are contained in a single cluster. Members of GH family 18 are separated into five clusters. One singleton, a GH 48 family protein belonging to *Ph. kernoviae*, is present in the network. Nodes are colored by order. An interactive version of this network is available online at https://oomycetes.github.io/chitinases.html. Download FIG S2, PDF file, 0.2 MB. Copyright © 2017 McGowan and Fitzpatrick. 2017 McGowan and Fitzpatrick This content is distributed under the terms of the Creative Commons Attribution 4.0 International license.

Our analysis also revealed 31 *Ap. astaci* proteins with N-terminal chitin-binding domains (Table S3); 25 (81%) of these were predicted to be secreted (Table S3). Other oomycetes contained fewer than 10 of these proteins, with *Al. candida*, *Al. laibachii*, *Ap. invadans*, and *Ph. rubi* containing no proteins with this domain. BLAST homology searches of these proteins against the NCBI databases did not reveal any hits with annotated proteins. Their function is unknown; it is possible that the N-terminal domain facilitates attachment to the chitin exoskeleton of its crayfish host.

### Proteases.

A large number of proteins with hydrolytic activity were reported, including glycoside hydrolases, proteases, and pectin modifying proteins (Table S3). These are thought to be involved in the degradation of host cells (8). Several protease families were found to make up a large part of oomycete secretomes, including aspartyl proteases, papain family cysteine proteases, subtilases, trypsins, and trypsin-like proteases. Members of the *Saprolegniales* order contained the largest number of proteases, with each member possessing over 150 proteins with predicted protease activity (Table S3). *Pythium* species also had a large number of proteases, which may be attributable to their highly damaging necrotrophic lifestyles. *Pythium* species had an average of 139 proteases, higher than the *Phytophthora* average of 88 (Table S3). A large number of these proteases were reported to be secreted in our SignalP analysis, indicating that they may be involved in the breakdown of host cells. For example, *Saprolegnia* species have an average of 86 secreted proteases, *Pythium* species have an average of 55, and *Phytophthora* have an average of 24 (Table S3). Obligate biotrophs such as *Al. laibachii*, *Al. candida*, and *H. arabidopsidis* feature the fewest proteases, with each species possessing fewer than 60 proteases. Expansions of aspartyl proteases were detected in *Py. insidiosum* (95 proteases), *Ph. ramorum* (70 proteases), and *Ph. sojae* (68 proteases). *Ap. astaci* contains 161 proteins with predicted trypsin or trypsin-like activity, the largest number of any species in our data set. SignalP predicted 93 (58%) of these to be secreted (Table S3). Every other oomycete had fewer than 61 copies. Our analysis also identified an expansion of proteins with subtilase domains in *Aphanomyces*, *Pythium*, and *Saprolegnia* species. Again, *Ap. astaci* contained a large repertoire of 94 proteins with subtilase domains compared to *Phytophthora* species, all of which has fewer than 15 subtilases (Table S3). Our results show that the *Ap. astaci* proteome harbors vast number of proteases, significantly more than any other oomycete. The large arsenal of hydrolytic enzymes in *Ap. astaci*, and in other *Saprolegniales* members, may play an important role in the degradation of host cells.

### Oomycete pectin modifying proteins.

Pectin is a major component of plant cell walls, making up to 35% of primary walls in higher plants, and plays important roles in plant defense, development, and growth (63). A total of 1,048 oomycete proteins were found to contain domains that are involved in the modification or degradation of pectin, including 693 pectate lyases, 226 pectin esterases, and 129 pectin acetylesterases (Table S3). Pectate lyases are involved in the cleavage of pectin and result in fruit softening and rot via degradation of the plant cell wall (64). Proteins with pectate lyase domains were abundant in *Phytophthora* and *Pythium* species (Table S3). They are completely absent in *Al. laibachii*, *Ap. astaci*, and *Ap. invadans*. One copy was found in each of *Al. candida*, *S. diclina*, and *S. parasitica*, but the *S. diclina* copy was not predicted to be secreted. Pectinesterases, or pectin methylesterases, catalyze the de-esterification of pectin and are used by plants in a wide range of biological processes, including cell wall remodeling, fruit ripening, pollen growth, and root development (65). However, they can also be exploited by pathogens to invade plant tissues (66). Our results show that pectin esterases were present only in members of the *Peronosporales* order in our data set (Table S3). The majority of these proteins contain signal peptides (Table S3), indicating that they are effectors that potentially cause damage to the cell walls of their hosts. Similarly, pectin acetylesterases can be exploited by pathogens to catalyze the deacetylation of pectin, making the pectin backbone more accessible to pectin-degrading enzymes such as pectate lyases (67). Proteins with the pectin acetylesterase domain were found in *Phytophthora*, *Phytopythium*, *Pilasporangium*, *Plasmopara*, and *Pythium* species (Table S3). Species with more pectate lyases typically had more pectin acetylesterases, and the correlation was strong and significant (*R*^2^ = 0.600, *P* < 0.00001 [Pearson correlation test]).

### Cutinases.

Cutin is one of the main components of plant cuticles (68). The plant cuticle acts as a physical barrier, allowing plant cells to tolerate external environmental stresses and also protecting interior plant tissues from invading pathogens (69). Cutinases are extracellular enzymes that hydrolyze cutin and have been identified in bacteria, fungi, and oomycetes (70). They can be used by plant pathogens to compromise the structural integrity of the plant cuticle, allowing them to penetrate and infect inner plant tissues. Enzymatic digestion of cutin has been proven to be an essential initial step in the infection process of plant pathogens (71).

Overall, we have identified 122 proteins with cutinase domains in our data set, 79 (65%) of which were predicted to be secreted (Table S3). Cutinases were completely absent in *Aphanomyces* and *Saprolegnia* species. *Pythium* species have previously been reported to lack cutinases (72). We found this to be true for the majority of *Pythium* species in our data set; however, we identified nine cutinases in *Py. aphanidermatum* and seven cutinases in *Py. arrhenomanes* (Table S3). Both *Albugo* species in our data set also contained multiple copies. *Ph. pinifolia* was the only *Peronosporales* member to lack cutinases.

### Toxin families.

We identified a number of toxin families in our data set, including necrosis-inducing proteins (see previous section) and members of the phytotoxic protein family (PcF). Relative to the level seen with other effectors, the overall number of PcFs detected in our data set was low. PcFs are known to induce necrosis (73). In total, 31 proteins with signatures of PcF proteins were identified and were unique to several members of the *Peronosporales*; 19 (61%) of these were predicted to be secreted (Table S3). *Ph. infestans* contains 14 PcF proteins, 5 of which are predicted to be secreted (Table S3). *Ph. capisci* contains six PcF proteins; *Ph. sojae* has four; *Ph. parasitica* has two; and *Ph. multivora*, *Ph. pisi*, and *Ph. ramorum* have one copy each. All of these are predicted to be secreted (Table S3). The proteome of *Ph. lateralis* was also reported to have one PcF protein, but it is not predicted to be secreted. *H. arabidopsidis* was the only non-*Phytophthora* species reported to have a PcF protein (Table S3). However, it was not predicted to be secreted. This finding suggests that PcF proteins are unique to *Phytophthora* and closely related species, suggesting that they may have arisen in the last common ancestor of the *Peronosporales* order.

### Crinklers.

A combination of regular expression searches and hidden Markov models was utilized to identify Crinkler effectors (CRNs). After manual inspection and removal of suspected false positives, a total of 899 CRNs were identified in our data set. Our results highlight that *Phytophthora* species possess large expansions of CRNs, more than the species of any other genera. In particular, we found 91, 92, and 167 CRNs in *Ph. capsici*, *Ph. sojae*, and *Ph. infestans*, respectively (Table S3). *Pilasporangium apinafurcum* and *Pl. viticola* also feature large numbers of CRNs relative to other species, having 52 and 65 CRNs each, respectively. CRN numbers were sparse in the *Albuginales* and *Saprolegniales* orders, with some species (*Ap. invadans*, *S. diclina*, and *S. parasitica*) possessing only one copy (Table S3). Thus, it would appear that CRNs play an important role in plant infection for *Phytophthora* species. Only 177 CRNs were predicted to be secreted in our SignalP analysis (Table S3). However, previous reports have indicated that a large number of CRNs may be secreted via unconventional protein secretion systems that cannot be predicted *in silico* (74).

### Oomycete protease inhibitors.

Plants and plant pathogens are constantly undergoing an evolutionary arms race with one another (75). Production and secretion of proteases by plants to degrade pathogen effectors are among the examples of this. To counteract this, plant pathogens, including oomycetes, have coevolved to secrete protease inhibitors (4). These protease inhibitors block the defensive mechanism of plant proteases. We have identified a number of secreted oomycete protease inhibitors. The most abundant were Kazal-type protease inhibitors (507 in total; 355 were predicted to be secreted) and cathepsin propeptide inhibitors (155 in total; 95 were predicted to be secreted) (Table S3). Counts of cathepsin propeptide inhibitors were consistent across our oomycete data set, with most species possessing fewer than 5 copies (Table S3). Kazal-type protease inhibitors were more abundant and not evenly distributed; for example, expansions were recorded in several species, including *Py. insidiosum* (43 copies), *Ph. sojae* (39 copies), and *Ph. infestans* (33 copies). Most other species contained fewer than 15 Kazal-type protease inhibitors. *Albugo* species have fewer than five copies each (Table S3).

### RxLR effectors.

Numerous *in silico* analyses performed using various bioinformatics strategies have been employed in previous studies to identify candidate RxLR effectors in oomycetes. The most liberal, first described by Win et al., is a strategy in which all possible open reading frames (ORFs) are examined for the presence of a signal peptide within the first 30 amino acid residues followed by an RxLR motif between residues 30 and 60 (Win method) (76). Extensions to this method have been developed (27, 76) with searches for a downstream EER motif (or a loose match); the EER motif is present in numerous validated RxLR effectors (regular expression or Regex) (27). Hidden Markov model (HMM) profiles derived from alignments of RxLR-EER effectors have also been implemented successfully (27). The initial genome analyses that described the *Ph. infestans* RxLR complement utilized all three approaches described above as well as additional criteria such as exhibiting sequence homology to previously known RxLRs or belonging to protein families where the majority of proteins are deemed putative RxLRs based on Win, Regex, or HMM criteria. Depending on the method or combination of methods utilized, it is possible to detect a broad range of potential RxLR effectors (8). As RxLR effectors are most abundant and have been best characterized in *Phytophthora* species (8), a *Phytophthora*-biased approach was taken to identify candidate oomycete RxLR effectors. To be classified as a putative RxLR effector, proteins or ORFs had to meet one of the RxLR criteria in analyses performed using the Win method, HMMsearch, Regex, or the Homologous method (see Materials and Methods).

The four RxLR criteria were tested on the predicted proteomes of each of the 37 oomycete species in our data set. Utilizing predicted proteins adds an additional criterion layer but may potentially miss open reading frames that were missed by gene callers during annotation. Additional criteria utilized by others such as belonging to a TribeMCL cluster (8) were not considered due to the associated computational costs. It is obvious that some of the criteria described above may detect a large number of false positives. For example, a number of the 563 ORFS counted as putative RxLRs in *Ph. infestans* were found by Blast homology alone and do not contain a RxLR domain; using these as “bait” in a BlastP search against other oomycetes will locate non-RxLR domain-containing homologs. However, we have noticed that when the RxLR repertoires of model genomes such as *Ph. infestans*, *Ph. sojae*, and *Ph. ramorum* are referenced in the literature, the researchers normally consider homologs of known RxLRs located via BlastP alone to represent putative RxLRs. Our analysis extends this as we use all proteins from these model genomes as bait sequences in our analysis. We also searched for the presence of repeating sequence motifs termed “W,” “Y,” and “L” that are found toward the C terminus of a number of *Phytophthora* cytoplasmic effectors (31). These domains form an alpha-helical fold known as the WY fold that may provide structural flexibility (32). However, due to the sequence divergence observed in *Phytophthora* RxLRs, it seems that alternative folds most likely exist (77).

Putative RxLRs along with sequence information and the criteria that were satisfied in classifying the proteins as putative RxLRs are listed in Table S6. For completeness and to allow comparisons to previous analyses, we also searched all putative ORFs that were nonoverlapping and more than 70 amino acids in length using the criteria described above (Table S7). We do not discuss the results of these counts here and instead concentrate on counts related to predicted protein coding genes. The purpose of this is to maintain consistency, as our secretome analysis also considered putative protein coding genes and did not look at all possible open reading frames.

**FIG 5:**
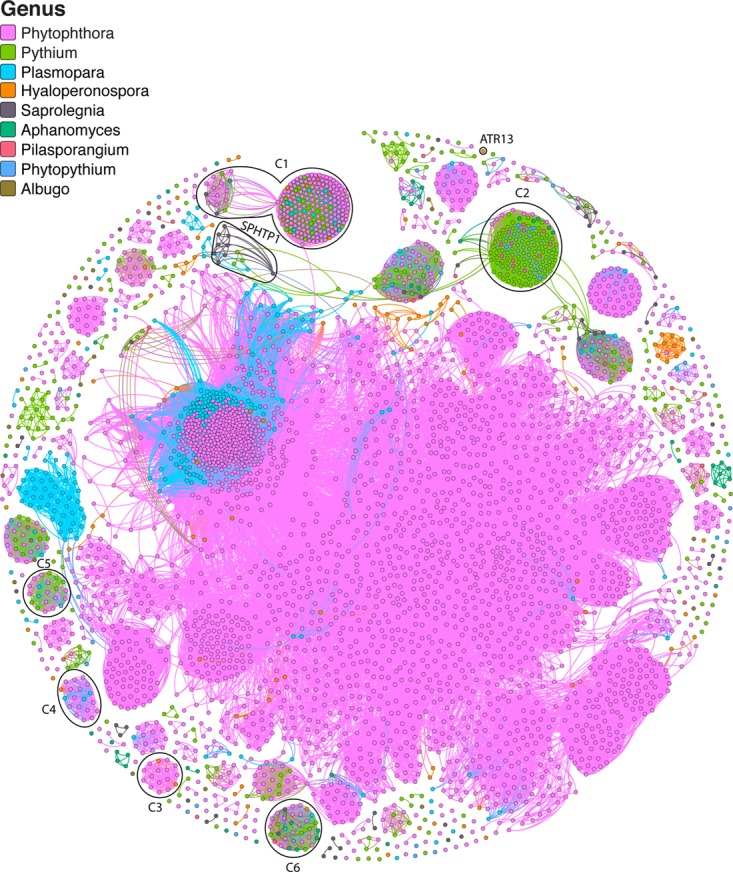
Homology network of 4,131 putative oomycete RxLR effectors. Each protein is represented by a node. An edge joining two nodes represents sequence similarity shared by the two proteins (*E* value cutoff of 10^−10^). Many disconnected clusters of RxLR effectors are represented, indicating no significant homology between clusters. Nodes are colored by genus. Clusters discussed in the text are labeled. An interactive version of this network is available online at https://oomycetes.github.io/rxlrs.html.

In total, 4,131 proteins in our data set matched one or more of the RxLR criteria, ranging from a low of 6 proteins in *Al. candida* to a high of 603 in *Ph. infestans* (Table S5). Unsurprisingly, the vast majority (3,600 or 87%) of the 4,131 proteins are located in *Peronosporales* species (Table S5). A homology network of the RxLR-like proteins was generated to investigate the evolutionary relationships within the putative RxLRs (Fig. 5). An interactive and searchable version of the network is available online at https://oomycetes.github.io. The online version of the network permits users to query the network based on the protein identifier (ID) and to retrieve sequence information as well as performing BlastP searches against the NCBI database. Furthermore, users can filter proteins based on genus or the RxLR criteria used (Win, Regex, HMM, or BLAST). Proteins can also be viewed based on the presence or absence of the WY fold. The network is composed of 4,131 nodes; each node corresponds to an individual protein and 184,302 edges; edges link nodes if they are homologous. The putative RxLRs were clustered into 357 connected components (Fig. 5). The nodes had an average degree of 89 (i.e., on average, each protein in the network has 89 homologs). The fact that the network is comprised of a number of disconnected clusters (individual clusters of connected nodes that are not connected to other clusters) shows there are no significant sequence similarities between some clusters (Fig. 5).

10.1128/mSphere.00408-17.8 TABLE S5  Overall counts (4,131) of putative RxLR proteins detected per species. Proteins detected by Win, Regex, HMM, or BLAST are given. Column G gives counts for proteins per species that have been detected by Win, Regex, or HMM (therefore, proteins found by homology searches alone). This count also omits proteins that were reported to contain a putative retention motif. Download TABLE S5, XLSX file, 0.05 MB. Copyright © 2017 McGowan and Fitzpatrick. 2017 McGowan and Fitzpatrick This content is distributed under the terms of the Creative Commons Attribution 4.0 International license.

10.1128/mSphere.00408-17.9 TABLE S6  List of all putative RxLR proteins detected in this analysis. Sequence, RxLR position, and cleavage site data are also included. The methodology used for labeling the protein as a putative RxLR is given in columns E to H. Download TABLE S6, XLSX file, 1.2 MB. Copyright © 2017 McGowan and Fitzpatrick. 2017 McGowan and Fitzpatrick This content is distributed under the terms of the Creative Commons Attribution 4.0 International license.

10.1128/mSphere.00408-17.10 TABLE S7  List of all putative RxLR ORFs detected in this analysis. Sequence, RxLR position, and cleavage site data are also included. The methodology used for labeling the ORF as a putative RxLR is given in columns C to G. Download TABLE S7, XLSX file, 2.7 MB. Copyright © 2017 McGowan and Fitzpatrick. 2017 McGowan and Fitzpatrick This content is distributed under the terms of the Creative Commons Attribution 4.0 International license.

**FIG 6:**
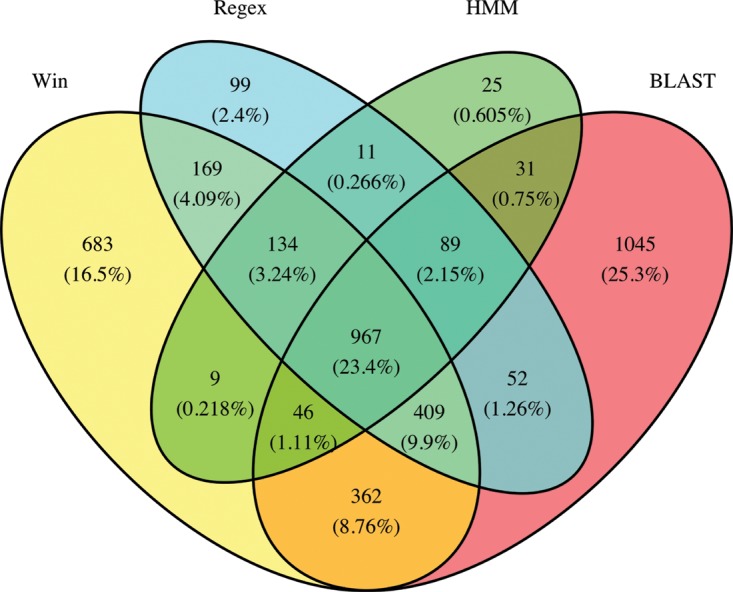
Venn diagram showing the overlap of the 4 categories of RxLR criteria (Win, Regex, HMM, and Blast) used in this analysis. A total of 967 *Phytophthora* proteins are labeled putative RxLRs based on all 4 criteria.

A significant proportion (1,852 or 44.8%) of the 4,131 proteins have only one line of evidence labeling them as RxLRs. For example, 1,045 (25.3%) were located based on BlastP homology alone (therefore, the RxLR domain is absent), 683 (16.5%) based on the Win method alone, 25 (0.6%) based on the HMM alone, and 99 (2.4%) on Regex alone (Fig. 6 and Fig. S3). Of the 1,045 proteins that were located by sequence homology exclusively (no evidence of RxLR motif), 981 were found in species from the *Peronosporales* order and may not be functional RxLRs, although 287 of these homologs were found to contain the WY fold (Table S6). Conversely, 967 (23.4%) of all proteins met the four RxLR criteria and all of these were *Phytophthora* proteins (Fig. 6; see also Fig. S3 and Table S6). Furthermore, 401 of these 967 proteins also contained the WY fold. Overall, of the 4,131 proteins, 1,123 were found to contain the WY fold and all proteins were from *Peronosporales* species (Table S6).

10.1128/mSphere.00408-17.3 FIG S3  Homology network of 4,131 putative oomycete RxLR effectors. The figure is identical to Fig. 5 except that the nodes are colored with respect to the number of RxLR criteria that labeled a particular node as being a putative RxLR. An interactive version of this network is available online at https://oomycetes.github.io/rxlrs.html. Download FIG S3, JPG file, 2 MB. Copyright © 2017 McGowan and Fitzpatrick. 2017 McGowan and Fitzpatrick This content is distributed under the terms of the Creative Commons Attribution 4.0 International license.

On closer examination of the similarity network and the criteria responsible for labeling proteins as putative RxLRs, is obvious that a number of clusters are potentially false positives. For example, cluster 1 (C1) contains a number of *Peronosporales* and *Pythiales* proteins; excluding a single edge corresponding to a large *Phytophthora* cluster, it represents a distinct cluster which shares no similarity with any other cluster in the network. The vast majority of proteins in this cluster have been labeled putative RxLRs based on BlastP alone (Fig. S3 and Table S6).

Similarly, C2 is composed of proteins that are also mostly from *Pythium* species (Fig. S3). All of these proteins have been labeled RxLR proteins based on the Win method exclusively. C3 is composed of *Peronosporales* proteins only, but all of these have been detected using BlastP alone (Fig. S3). There are many other examples of potentially false-positive RxLR clusters, including C4 (detected using Win and BlastP) and C5 (detected using Win exclusively) (Fig. S3 and Table S6). Note, however, that the RxLR search performed here labeled a *S. parasitica* protein (SAPA|10778) a putative RxLR. This putative RxLR effector protein (SPHTP1) has been shown to be translocated into fish cells and may play an important role in saprolegniosis (78). The uptake of SPHTP1 is mediated by an interaction with tyrosine-O-sulfate-modified cell surface molecules (79) and not via phospholipids, as is the case for RxLR effectors from oomycete plant pathogens (80). Examining our RxLR network, we see that SPHTP1 lies in a cluster which is composed primarily of *Saprolegnia* proteins, with some of these proteins having homology to *Plasmopara* and *Pythium* proteins (Fig. 5). *S. parasitica* contains 5 homologs of this protein, while *S. diclina* contains 3. Similarly, our RxLR analysis labeled a *H. arabidopsidis* protein (HYAP|12966 or ATR13) a putative RxLR, again based on the Win method alone. ATR13 was found as a singleton with no homology to any other putative RxLR in our network, which is unsurprising, as high levels of polymorphism have been reported for this protein. It has been shown that ATR13 has the ability to translocate into host cells (81). Similarly, another previously described RxLR effector from *H. arabidopsidis*, ATR1 (HYAP|01864), was also labeled a putative RxLR based on Win and Regex criteria (Table S6).

C6 contains 33 RxLRs, with proteins from every genus in our data set except *Albugo*. All of these proteins have homology to reference RxLRs (Table S6) and are classified RxLRs based on the Win or Regex criteria. The group forms a clique (a subnetwork where each member is connected to every other member), showing that every protein in the group is homologous to every other protein. On average, these proteins have sequence similarity of 52%. The position of the RxLR motif is ubiquitously conserved (not shown). However, the majority of these proteins contain a KDEL endoplasmic reticulum (ER) retention motif at the C terminus as previously reported for the *Py. oligandrum* protein represented in this cluster (52). Proteins with this ER motif are not predicted to be secreted and therefore likely represent false positives (52). Overall, 75 of the 4,131 putative RxLRs contain the KDEL motif (or a variation) (Table S6).

Of the 4,131 putative RxLRs, 3,011 were located based on Win, Regex, or HMM results, meaning that 1,120 have been labeled due to homology to a reference RxLR alone, or they may also contain a KDEL retention motif and so may not be secreted. Ignoring these 1,120 proteins, we observe that *Ph. infestans* is predicted to contain 470 RxLRs, a figure that is consistent with the figure of 563 putative RxLRs reported based on analysis of ORFs (8). Furthermore, 95 of these 563 ORFs were labeled based on homology alone, therefore giving an overall number of 468 if non-RxLR-containing homologs are excluded. Similarly, the comparison between our *Ph. sojae* RxLR prediction number and the corresponding number reported from previous studies (338 versus 312) is consistent.

### Conclusions.

The first oomycete genome sequences were published in 2009 (8), and at the time of writing there were 37 oomycete genome assemblies publically available. Due to their importance as pathogens of economically important crops and animals, along with the ongoing advances and reductions in costs associated with next-generation sequencing technologies, this number is expected to increase dramatically over the coming years. In this analysis, we performed an inventory of known oomycte effectors in all available genome sequences. As well as quantifying their occurrences, we have in a number of cases also investigated their evolutionary history.

This genome-wide survey provides an up-to-date inventory of previously described effectors in the class *Oomycota*. It is by no means a complete list, and we are cognizant that many additional effectors will be characterized in the coming years, especially with improved host-pathogen interaction omics studies. However, it does provide the current overview of the arsenal of known effectors in this economically important class of animal and plant pathogens.

We have also examined the presence and absence of glycoside hydrolases and found a diverse range of families across the oomycote class. The majority (54%) of these are secreted. Glycoside hydrolase families 18, 19, and 48 all have chitinase activity. A homology network (Fig. S2) showed that there is no sequence homology between family members 18 and 19. Interestingly, family 18 can be subdivided into 4 distinct clusters, indicating that while family members have the same enzymatic function, they do not share sequence similarity, confirming that family 18 has different subclasses. Our analysis has also detected the presence of immunoglobulin A peptidases in *Pythiales* species and *Aphanomyces* species (Fig. 4). Some of these species are animal pathogens, and it is possible that their presence may be important in suppressing the immune response of their animal hosts.

We have also catalogued the putative RxLR repertoire of all 37 oomycete species. Our results are consistent with those of other studies in showing that *Phytophthora* species have undergone expansions in these proteins. We are aware that this analysis may be reporting false positives, particularly for species outside the *Peronosporales* order. However, a number of previously described RxLR effectors such as *H. arabidopsidis* ATR1 and ATR13 were detected, as was SPHTP1 *S. parasitica*. We are also aware that we may be underreporting the number of RxLRs, as we did not analyze all possible open reading frames and instead concentrated on predicted protein coding genes; this decision was taken because our secretome analysis also used protein coding genes. Furthermore, using open reading frames could in itself lead to the reporting of false positives by reporting pseudogenes or noncoding regions of the genome. The confirmation of all putative RxLRs is beyond the scope of this *in silico* catalogue; however, detailed sequence information on all proteins is provided in the supplemental material.

## MATERIALS AND METHODS

### Data set assembly.

The predicted proteomes for 23 oomycete species were obtained from public databases (Table 1). Predicted proteomes for another 14 oomycete species (10 *Phytophthora* species and *Pl. viticola*, *Pi. apinafurcum*, *Py. insidiosum*, and *Py. oligandrum*) were generated from publically available data using AUGUSTUS (82) (Table 1). Templates for *ab initio* protein prediction were generated for AUGUSTUS using assembly and expressed sequence tag (EST) data from a number of reference oomycete species. *Ph. capsici* was used as a reference for *Ph. agathidicida*, *Ph. multivora*, *Ph. pluvialis*, and *Ph. taxon totara*. *Ph. sojae* was used as a reference for *Ph. cinnamomi*, *Ph. cryptogea*, *Ph. fragariae*, *Ph. pinifolia*, *Ph. pisi*, and *Ph. rubi*. *Pl. halstedii* was used as a reference for *Pl. viticola*. *Py. ultimum* var. *sporangiiferum* was used as a reference for the two *Pythium* species. GeneMark-ES (83) was used in addition to AUGUSTUS for predicting proteins of *Pi. apinafurcum*. The final data set contained 590,896 proteins from 37 predicted oomycete proteomes (Table 1). All proteomes used in this analysis as well as pipelines and scripts for identification of effectors, secretomes, and RxLRs are available at https://github.com/oomycetes/oomycetes.github.io/tree/master/SupplementaryMaterial.

### Identification of putative effectors.

InterProScan 5 (41) was run on all 590,896 predicted oomycete proteins in our data set. Any proteins reported by InterProScan as having a Pfam domain that could be implicated in pathogenesis were classified as potential effectors (see Table S3 in the supplemental material). The list of pathogenic Pfam domains considered was based on a number of previous analyses (14, 26, 37).

### Identification of putatively secreted proteins.

Transmembrane domain prediction was carried out for all 590,896 proteins using THMM (84), and signal peptides were predicted using SignalP v3 (85). Proteins that had an HMM S probability value of ≥0.9, an NN *Y*_max_ score of ≥0.5, and an NN *D* score of ≥0.5 with predicted localization “Secreted” and no transmembrane domain after the signal peptide cleavage site were considered to be putatively secreted.

### Enrichment analyses.

Enrichment for particular Pfam and GO terms was undertaken in Blast2GO (86) by comparing the frequencies of GO terms and Pfam domains in the secretome to those in the nonsecreted proportion of the proteome using Fisher's exact test corrected for false-discovery rate (FDR) (40). Only Pfam domains or GO terms with enrichment *P* values of <0.05 are reported.

### Identification of putative RxLR effectors.

Proteins were classified as putative RxLR effectors if they satisfied one of the following four criteria. (i) In the Win method, the protein must contain a signal peptide in residues 1 to 30 followed by an RxLR motif within residues 30 to 60 (76). (ii) In the HMMsearch method, hidden Markov model analysis was performed for all proteins predicted to be secreted to detect the RxLR motif using the "cropped.Hmm" HMM profile constructed by Whisson et al. (27). This accounts for variations in the RxLR and EER motifs. Matches with a bit score of >0 were retained. (iii) In the Regex method, the protein must contain a signal peptide between residues 10 to 40 and an RxLR motif within the following 100 residues followed by the EER motif within 40 residues downstream of the RxLR motif (allowing replacements of E to D and R to K) (27). The regular expression used was as follows: ^.{10,40}.{1,96}R.LR.{1,40}[ED][ED][KR]. (iv) In the Homologous method, the set of 1,207 putative *Phytophthora* RxLR effectors was downloaded for *Ph. infestans*, *Ph. ramorum*, and *Ph. sojae* (8). These were used as reference RxLRs in the RxLR homology search. Proteins located via a BlastP search (*E* value cutoff, 10^−20^) corresponding to a reference *Phytophthora* RxLR were considered putative RxLRs.

A HMM search was run on all candidate RxLRs to determine if they contain the WY fold using the HMM method developed by Boutemy et al. (77).

### Identification of Crinkler effectors.

String searches were performed to account for variations in the Crinkler “LxLFLAK” motif. First, a search was carried out with the regular expression "^.{30,70}LFLA[RK]." All hits were aligned using MUSCLE (v3.8.31) (87). A hidden Markov model (HMM) was built for the alignment using HMMER (3.1) (88). The HMM was searched against our entire data set using hmmsearch to identify homologs. A second string search was carried out using the regular expression "^.{30,70}LYLA[RK]." Again, hits from this search were aligned using MUSCLE and an HMM was built and searched against our data set. The two results were merged as our candidate Crinkler effector set. These candidates were manually inspected, and any proteins that did not contain an obvious “LxLFLAK-like” motif were excluded.

### Generation of homology networks.

Homology networks were generated for a number of protein families. In each instance, an all-versus-all BLASTp search (89) was run against each member of the family with an *E* value cutoff of 10^−10^ for the chitinase and NLP networks and an *E* value cutoff of 10^−5^ for the RxLR network. Each protein was represented by a node in the network. Two proteins were connected by an undirected edge if they were identified as homologous in our all-versus-all BLASTp search. The network was visualized and annotated in Gephi (90) and arranged using the Fruchterman-Reingold layout (91). Network statistics were calculated within Gephi. Online interactive versions of all networks are available at https://oomycetes.github.io. Protein/node information is available for viewing in the network. The networks can be filtered to hide/show particular proteins by protein ID, species, genus, or order.

### Maximum-likelihood phylogenetic reconstruction of effector families.

For the NLP phylogeny, the 25 type 2 NLPs were used as bait sequences in a BLASTp (89) homology search (*E* value cutoff of 10^−20^) against a local database of 8,805,033 proteins (53). All homologs were aligned using MUSCLE (87) and manually edited, giving a final alignment of 460 amino acids. ModelGenerator inferred that the optimum model of substitution was the WAG model of substitution (92). A maximum-likelihood phylogeny was reconstructed in PhyML using this model, and 100 bootstrap replicates were undertaken (93). The final tree was visualized and annotated with iTOL (94).

For the IgA peptidase-containing proteins, all 40 were aligned using MUSCLE and edited to give a final alignment of 770 amino acids. ModelGenerator inferred that the optimum model of substitution was the WAG model of substitution. A maximum-likelihood phylogeny of all IgA peptidase-containing proteins was generated using a WAG substitution model in PhyML, and 100 bootstrap replicates were undertaken. The final tree was visualized and annotated with iTOL (94).
