# Supplementary material for: Genomic, Network, and Phylogenetic Analysis of the Oomycete Effector Arsenal
Source: mSphere. 2017 Nov 22;2(6):e00408-17. doi: 10.1128/mSphere.00408-17 (PMC5700374; doi:10.1128/mSphere.00408-17)
Supplement: FIG S1 [file sph006172413sf1.pdf]

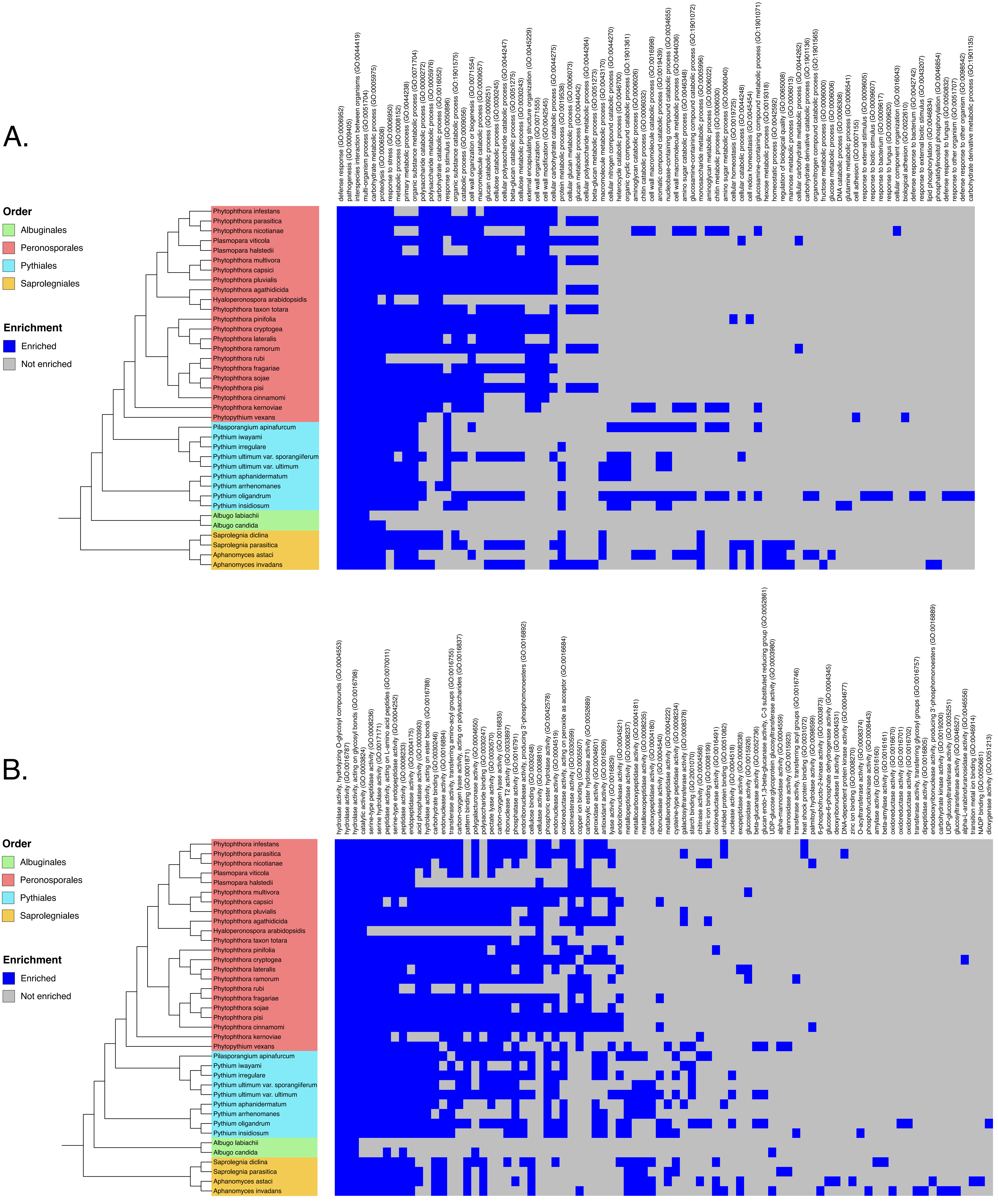

A.
defense response (GO:0006952)
pathogenesis (GO:0009405)
interspecies interaction between organisms (GO:0044419)
multi-organism process (GO:0051704)
carbohydrate metabolic process (GO:0005975)
proteolysis (GO:0006508)
response to stress (GO:0006950)
metabolic process (GO:0008152)
primary metabolic process (GO:0044238)
organic substance metabolic process (GO:0071704)
polysaccharide catabolic process (GO:0000272)
polysaccharide metabolic process (GO:0005976)
carbohydrate catabolic process (GO:0016052)
response to stimulus (GO:0050896)
organic substance catabolic process (GO:1901575)
catabolic process (GO:0009056)
cell wall organization or biogenesis (GO:0071554)
macromolecule catabolic process (GO:0009057)
glucan catabolic process (GO:0009251)
cellulose catabolic process (GO:0030245)
cellular polysaccharide catabolic process (GO:0044247)
beta-glucan catabolic process (GO:0051275)
cellulose metabolic process (GO:0030243)
external encapsulating structure organization (GO:0045229)
cell wall organization (GO:0071555)
cell wall modification (GO:0042545)
cellular carbohydrate catabolic process (GO:0044275)
protein metabolic process (GO:0019538)
cellular glucan metabolic process (GO:0006073)
glucan metabolic process (GO:0044042)
cellular polysaccharide metabolic process (GO:0044264)
beta-glucan metabolic process (GO:0051273)
macromolecule metabolic process (GO:0043170)
cellular nitrogen compound catabolic process (GO:0044270)
heterocycle catabolic process (GO:0046700)
organic cyclic compound catabolic process (GO:1901361)
aminoglycan catabolic process (GO:0006026)
chitin catabolic process (GO:0006032)
cell wall macromolecule catabolic process (GO:0016998)
aromatic compound catabolic process (GO:0019439)
nucleobase-containing compound catabolic process (GO:0034655)
cell wall macromolecule metabolic process (GO:0044036)
amino sugar catabolic process (GO:0046348)
glucosamine-containing compound catabolic process (GO:1901072)
monosaccharide metabolic process (GO:0005996)
aminoglycan metabolic process (GO:0006022)
chitin metabolic process (GO:0006030)
amino sugar metabolic process (GO:0006040)
cellular homeostasis (GO:0019725)
cellular catabolic process (GO:0044248)
cell redox homeostasis (GO:0045454)
glucosamine-containing compound metabolic process (GO:1901071)
hexose metabolic process (GO:0019318)
homeostatic process (GO:0042592)
regulation of biological quality (GO:0065008)
mannose metabolic process (GO:0006013)
cellular carbohydrate metabolic process (GO:0044262)
carbohydrate derivative catabolic process (GO:1901136)
organonitrogen compound catabolic process (GO:1901565)
fructose metabolic process (GO:0006000)
glucose metabolic process (GO:0006006)
DNA catabolic process (GO:0006308)
glutamine metabolic process (GO:0006541)
cell adhesion (GO:0007155)
response to external stimulus (GO:0009605)
response to biotic stimulus (GO:0009607)
response to bacterium (GO:0009617)
response to fungus (GO:0009620)
cellular component organization (GO:0016043)
biological adhesion (GO:0022610)
defense response to bacterium (GO:0042742)
response to external biotic stimulus (GO:0043207)
lipid phosphorylation (GO:0046834)
phosphatidylinositol phosphorylation (GO:0046854)
defense response to fungus (GO:0050832)
response to other organism (GO:0051707)
defense response to other organism (GO:0098542)
carbohydrate derivative metabolic process (GO:1901135)
Order
Albuginales
Peronosporales
Pythiales
Saprolegniales
Enrichment
Enriched
Not enriched
Phytophthora infestans
Phytophthora parasitica
Phytophthora nicotianae
Plasmopara viticola
Plasmopara halstedii
Phytophthora multivora
Phytophthora capsici
Phytophthora pluvialis
Phytophthora agathidicida
Hyaloperonospora arabidopsidis
Phytophthora taxon totara
Phytophthora pinifolia
Phytophthora cryptogea
Phytophthora lateralis
Phytophthora ramorum
Phytophthora rubi
Phytophthora fragariae
Phytophthora sojae
Phytophthora pisi
Phytophthora cinnamomi
Phytophthora kernoviae
Phytopythium vexans
Pilasporangium apinafurcum
Pythium iwayami
Pythium irregulare
Pythium ultimum var. sporangiiferum
Pythium ultimum var. ultimum
Pythium aphanidermatum
Pythium arrhenomanes
Pythium oligandrum
Pythium insidiosum
Albugo labiachii
Albugo candida
Saprolegnia diclina
Saprolegnia parasitica
Aphanomyces astaci
Aphanomyces invadans
B.
hydrolase activity, hydrolyzing O-glycosyl compounds (GO:0004553)
hydrolase activity (GO:0016787)
hydrolase activity, acting on glycosyl bonds (GO:0016798)
catalytic activity (GO:0003824)
serine-type peptidase activity (GO:0008236)
serine hydrolase activity (GO:0017171)
peptidase activity, acting on L-amino acid peptides (GO:0070011)
serine-type endopeptidase activity (GO:0004252)
peptidase activity (GO:0008233)
endopeptidase activity (GO:0004175)
acid phosphatase activity (GO:0003993)
hydrolase activity, acting on ester bonds (GO:0016788)
carbohydrate binding (GO:0030246)
endonuclease activity (GO:0016894)
transferase activity, transferring amino-acyl groups (GO:0016755)
carbon-oxygen lyase activity, acting on polysaccharides (GO:0016837)
pattern binding (GO:0001871)
polygalacturonase activity (GO:0004650)
polysaccharide binding (GO:0030247)
pectate lyase activity (GO:0030570)
carbon-oxygen lyase activity (GO:0016835)
ribonuclease T2 activity (GO:0033897)
phosphatase activity (GO:0016791)
endoribonuclease activity, producing 3'-phosphomonoesters (GO:0016892)
cellulose binding (GO:0030248)
cellulase activity (GO:0008810)
phosphoric ester hydrolase activity (GO:0042578)
endonuclease activity (GO:0004519)
oxidoreductase activity, acting on peroxide as acceptor (GO:0016684)
pectinesterase activity (GO:0030599)
copper ion binding (GO:0005507)
carboxylic ester hydrolase activity (GO:0052689)
peroxidase activity (GO:0004601)
antioxidant activity (GO:0016209)
lyase activity (GO:0016829)
endoribonuclease activity (GO:0004521)
metallopeptidase activity (GO:0008237)
metallocarboxypeptidase activity (GO:0004181)
metalloexopeptidase activity (GO:0008235)
carboxypeptidase activity (GO:0004180)
ribonuclease activity (GO:0004540)
metalloendopeptidase activity (GO:0004222)
cysteine-type peptidase activity (GO:0008234)
galactosyltransferase activity (GO:0008378)
starch binding (GO:2001070)
chitinase activity (GO:0004568)
ferric iron binding (GO:0008199)
oxidoreductase activity (GO:0016491)
unfolded protein binding (GO:0051082)
nuclease activity (GO:0004518)
exopeptidase activity (GO:0008238)
glucosidase activity (GO:0015926)
beta-glucanase activity (GO:0052736)
glucan endo-1,3-beta-glucanase activity, C-3 substituted reducing group (GO:0052861)
UDP-glucose:glycoprotein glucosyltransferase activity (GO:0003980)
alpha-mannosidase activity (GO:0004559)
mannosidase activity (GO:0015923)
transferase activity, transferring acyl groups (GO:0016746)
heat shock protein binding (GO:0031072)
palmitoyl hydrolase activity (GO:0098599)
6-phosphofructo-2-kinase activity (GO:0003873)
glucose-6-phosphate dehydrogenase activity (GO:0004345)
deoxyribonuclease II activity (GO:0004531)
DNA-dependent protein kinase activity (GO:0004677)
zinc ion binding (GO:0008270)
O-acyltransferase activity (GO:0008374)
phosphofructokinase activity (GO:0008443)
amylase activity (GO:0016160)
beta-amylase activity (GO:0016161)
oxidoreductase activity (GO:0016670)
oxidoreductase activity (GO:0016701)
oxidoreductase activity (GO:0016702)
transferase activity, transferring glycosyl groups (GO:0016757)
dipeptidase activity (GO:0016805)
endodeoxyribonuclease activity, producing 3'-phosphomonoesters (GO:0016889)
carbohydrate kinase activity (GO:0019200)
UDP-glucosyltransferase activity (GO:0035251)
glucosyltransferase activity (GO:0046527)
alpha-L-arabinofuranosidase activity (GO:0046556)
transition metal ion binding (GO:0046914)
NADP binding (GO:0050661)
dioxygenase activity (GO:0051213)
Order
Albuginales
Peronosporales
Pythiales
Saprolegniales
Enrichment
Enriched
Not enriched
Phytophthora infestans
Phytophthora parasitica
Phytophthora nicotianae
Plasmopara viticola
Plasmopara halstedii
Phytophthora multivora
Phytophthora capsici
Phytophthora pluvialis
Phytophthora agathidicida
Hyaloperonospora arabidopsidis
Phytophthora taxon totara
Phytophthora pinifolia
Phytophthora cryptogea
Phytophthora lateralis
Phytophthora ramorum
Phytophthora rubi
Phytophthora fragariae
Phytophthora sojae
Phytophthora pisi
Phytophthora cinnamomi
Phytophthora kernoviae
Phytopythium vexans
Pilasporangium apinafurcum
Pythium iwayami
Pythium irregulare
Pythium ultimum var. sporangiiferum
Pythium ultimum var. ultimum
Pythium aphanidermatum
Pythium arrhenomanes
Pythium oligandrum
Pythium insidiosum
Albugo labiachii
Albugo candida
Saprolegnia diclina
Saprolegnia parasitica
Aphanomyces astaci
Aphanomyces invadans
