# Supplementary figures and images for: Genomic, Network, and Phylogenetic Analysis of the Oomycete Effector Arsenal
Source: mSphere. 2017 Nov 22;2(6):e00408-17. doi: 10.1128/mSphere.00408-17 (PMC5700374; doi:10.1128/mSphere.00408-17)

## Order

- Albuginales
- Peronosporales
- Pythiales
- Saprolegniales

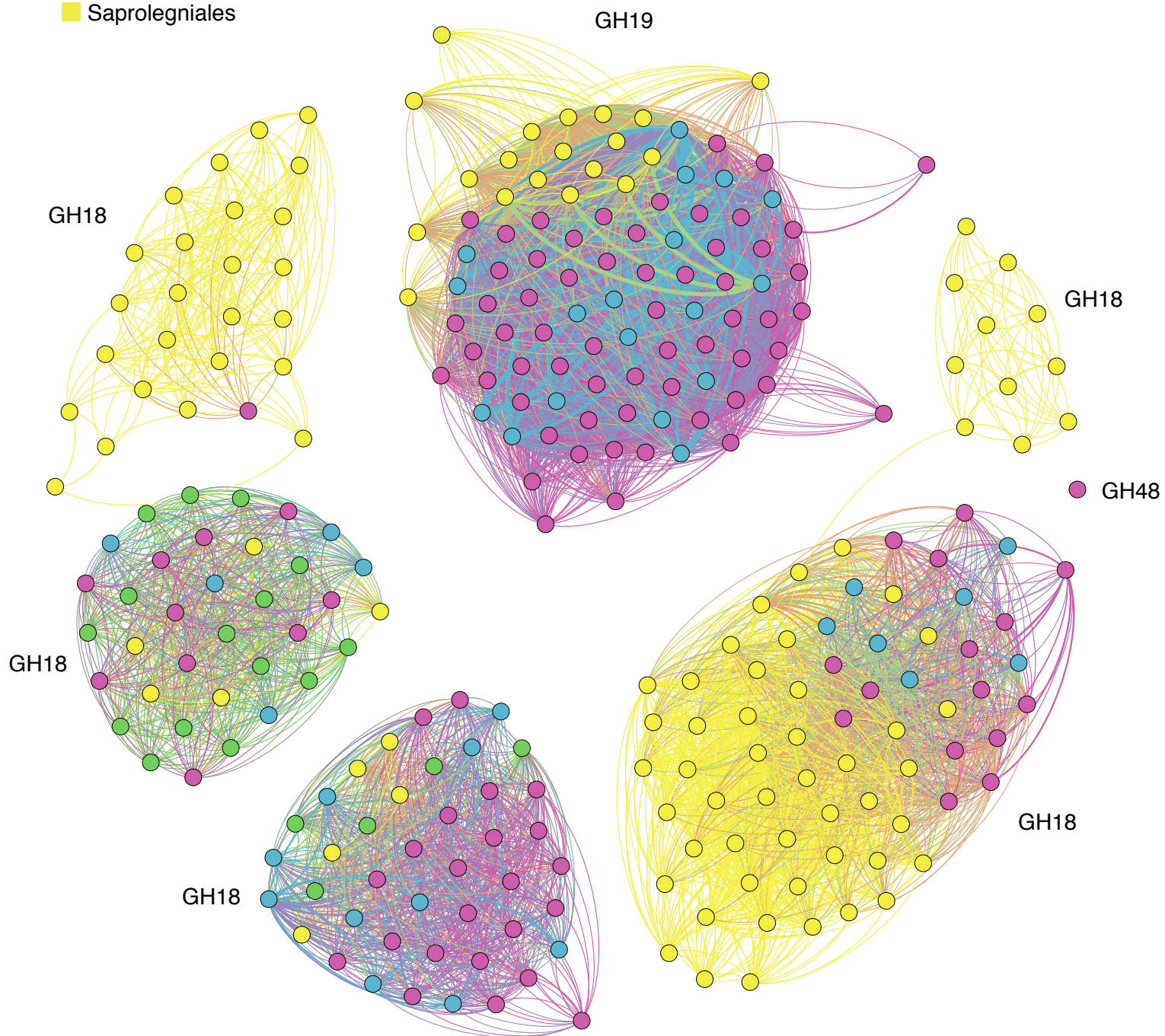

Supplement: FIG S2 [file sph006172413sf2.pdf]

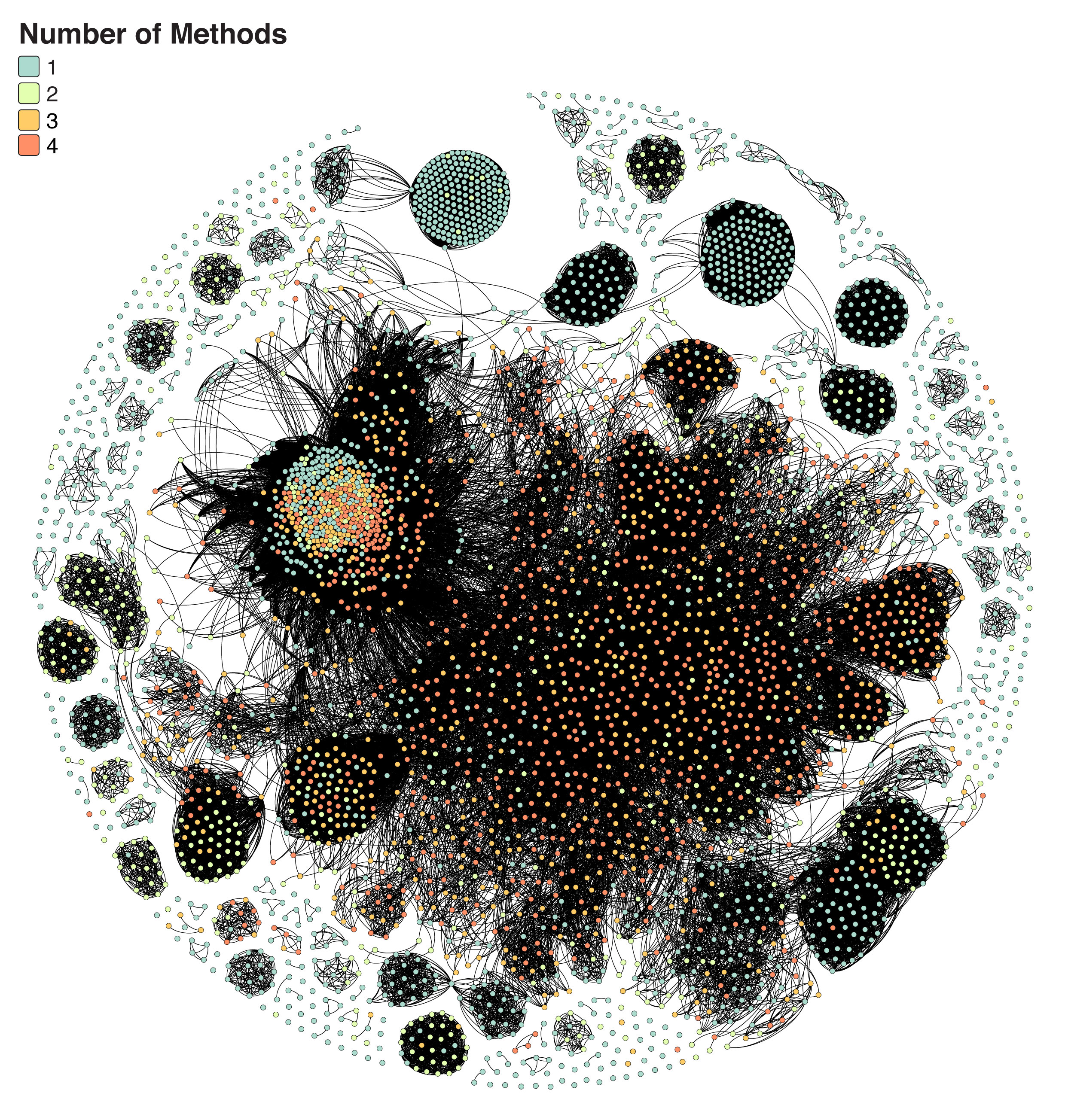

Supplement: FIG S3 [file sph006172413sf3.jpg]
